# Dental Caries Profile and Associated Risk Factors Among Adolescent School Children in an Urban South-Indian City

**DOI:** 10.3290/j.ohpd.a43368

**Published:** 2020-07-04

**Authors:** Poornima Reddy, Jogikalmat Krithikadatta, Valarmathi Srinivasan, Sandhya Raghu, Natanasabapathy Velumurugan

**Affiliations:** a Consultant Endodontist, Sharada Dental Hospital, Chittoor, India. Literature review, data collection and manuscript preparation.; b Associate Professor, Department of Conservative Dentistry and Endodontics, Faculty of Dentistry, MAHER, Chennai, India. Protocol development, literature review, statistical interpretation, manuscript preparation.; c Biostatistician. Department of Epidemiology, The Tamilnadu Dr. MGR Medical University, Chennai, India. Statistical analysis, statistical interpretation.; d Reader, Department of Conservative Dentistry and Endodontics, Saveetha Dental College and Hospitals, Chennai, India. Literature review and manuscript preparation.; e Head of Department, Department of Conservative Dentistry and Endodontics, Faculty of Dentistry, MAHER, Chennai, India. Literature review, manuscript preparation.

**Keywords:** adolescent children, dental caries, dental plaque, ICDAS, risk assessment, saliva

## Abstract

**Purpose:**

To study the dental caries experience among adolescent school children in Chennai city using the ICDAS-II scoring system. The secondary objective was to identify associated risk factors to different thresholds of dental caries defined by ICDAS.

**Material and Methods:**

Two hundred and thirty-seven children (13–17 years) from five schools across Chennai city were included using simple random sampling. After obtaining assent to participate in the study and satisfying the selection criteria, 200 children were screened for dental caries using ICDAS-II. The population was assessed for the following risk factors: sociodemographic status, habits, diet, plaque and salivary parameters. Prevalence of dental caries was estimated at the following thresholds: normal (ICDAS-0/1), mild caries (ICDAS-2), moderate caries (ICDAS-3/4) and extensive caries (ICDAS-5/6). Backward logistic regression analysis was performed to identify risk factors at different thresholds and crude odds ratio was calculated for statistically significant risk factors.

**Results:**

The overall prevalence of dental caries (ICDAS 3–6) was 57.5% (95% CI 48–62%). The proportions of children at different caries thresholds were: ICDAS-2 – 55% (95% CI:48–62%), ICDAS-3/4 – 51% (95% CI:44–58%) and ICDAS-5/6 – 25% (95% CI:19–31%). Reduced pH was statistically significant for moderate and extensive caries (OR 6.24, 95% CI 1.18–32.78 and 1.73, 95% CI 1.18–1.92, respectively) and the quantity of saliva was statistically significant for mild and moderate caries (OR 4.48, 95% CI 2.94–8.23 and 3.97, 95% CI 2.65–7.03, respectively). Low buffering capacity was associated with mild caries OR 5.71, 95% CI 2.82–18.2). Interobserver correlation was 0.91. A non-statistically significant value using Hosmer–Lemeshow Goodness of Fit test indicated that all three models predict the true estimate of the population.

**Conclusion:**

The proportions of children with mild and moderate caries were high considering their age group. The risk factors associated with mild caries were different from those associated with moderate and extensive caries.

Dental caries is the localised destruction of susceptible dental hard tissue by acidic by-products from bacterial fermentation of dietary carbohydrates.^25^ The infectious disease process can be arrested at any point in time. Dental caries has numerous and complex relations and interactions between the risk factors such as high sugar consumption, a shift in diet, brushing habits, socioeconomic status, the rate of urbanisation and the mother’s level of education.^10^ Conducive substrate, *Streptococcus mutans* and fermentable carbohydrates remain the primary reasons. Considering the multifactorial aetiology of caries, one should be aware of these risk factors associated with the disease so as to decrease its prevalence^9^ and studying these factors may help in the identification of populations or individuals at risk.^17^ Caries risk assessment tools can be used to determine an individual’s expected caries experience over a period of time and the likelihood of new caries activity and progression rate of existing carious lesions.^44^ The International Caries Detection and Assessment System (ICDAS) presents a new paradigm for the assessment of dental caries.^21^ The changing disease patterns with a general caries decline associated with a relatively high number of non-cavitated caries lesions was one of the reasons that led to the development of the harmonised International Caries Detection and Assessment System II (ICDAS-II) in recent years. Since ICDAS ascertains the severity of caries experience, it enables the designing of targeted care for the population.^24^

Cross-sectional studies play a major role in estimating the prevalence of the disease and identifying the population at risk. They also help in identifying the factors or indicators associated with caries status or severity of the disease.^22^ The prevalence studies across the world showed the prevalence rates as follows: Malaysia(30%),^27^ China (53%),^39^ and South Africa (46%),^6^ report greater caries prevalence, as compared to developed countries like England (32%),^32^ and Italy (22%).^5^ However in India, a cross-sectional study, Sarumathi et al, found the prevalence of dental caries among 3-year-old children to be 44.3%, 57.8% in 4 year olds, in 5-year-olds 72.0% and 74.1% in 6-year-olds in Chennai city, India.^20^ Simratvir et al studied 3–6-year-old children in Ludhiana and found 53% of them having dental caries.^42^ The continued high prevalence requires a periodic screening and risk assessment of subjects that will help in the development of better methods to identify individuals and tooth sites at risk for caries development. It also aids in planning primary prevention programmes to decrease the burden of disease which would otherwise disable the functional component of the oral cavity.^46^

 According to the World Health Organization (WHO), all permanent teeth would be erupted and erupted teeth would remain exposed to the risk factors over a considerable time period during age groups 13–17 years.^7^ The WHO has proposed that until 2020, the impact of oral and craniofacial illnesses on an individual’s health and psychosocial development should be decreased, emphasising the importance of the promotion of oral health and decreasing illnesses of the oral cavity, which are affected by diseases or disease-promoting conditions.^19^

Thus, updating the information on the prevalence of early caries lesions, its exact nature, and the degree of severity and understanding its association with the specific factors in age group of 13–17 years school children, are required for a better understanding of the current situation and for planning community caries prevention programmes. To our best knowledge, dental caries experience for adolescent age group, based on severity is not available for Indian population. Therefore, this study was undertaken to study the caries profile in adolescent school children in an urban Indian city.

## Materials and Methods

### Study Population

A cross-sectional survey was carried out on both male and female children aged 13–17 years selected from five private schools at Chennai, that represented varied socioeconomic strata. The study was conducted between June to July 2014. Ethical clearance for this study was obtained from MAHER University (MADC/IRB/2014/026). Stratified random sampling of 50 children from each of the five schools studying in standards ninth to twelfth were invited to participate in the study.

### Inclusion and Exclusion Criteria

Children who were natives of Chennai city, and were willing to participate in the study with concomitant assent from parent were included in the study. Students not willing to participate in the study, those with mixed dentition, fixed orthodontic appliances, teeth with fluorosis or with systemic illnesses were excluded. Out of 234 subjects who were given the assent form, 220 subjects were willing to participate in the screening. Based on the selection criteria 200 students of age group 13–17 years were included in the study for further evaluation.

### Questionnaire

A structured, close-ended questionnaire was constructed by consolidating several parameters detailed in various risk assessment systems/guidelines (American Academy of Paediatric Dentistry (AAPD),^1^ Caries Management by Risk Assessment (CAMBRA),^14^ American Dental Association (ADA),^2^ Cariogram^3^) and translated into the local language. The feasibility of measuring these risk factors in our population was assessed initially through focus group discussion and subsequently through a pilot study. The factors studied were included in three categories, namely; protective factors, risk factors and disease indicators. Use of fluoridated toothpaste, frequency of brushing, use of mouthwash and chewing gum were measured under protective factors. The risk indicators included employment and education of the parents, type of school, last dental visit, diet, frequency of snacking and consumption of sugary foods. Under disease indicators, the plaque scores, salivary parameters and severity of dental caries were measured.

### Data Collection

#### Sociodemographic and behaviour details

Sociodemographic details of parent employment and education; and type of school were collected from the questionnaire. Also, details of personal behaviour and dental habits were noted.

#### Salivary parameters

Individual cross-infection protection measures were employed. A portable source of operating light, packaged and sterilised mouth mirrors and WHO probe (CP-11.5B screening colour-coded probe, Hu-Friedy) were used for the examination. Unstimulated saliva was allowed to pool in the subject’s mouth for 1 min and consistency was noted. Buffering capacity was assessed using a calorimetric strip test (Saliva-buffer Check kit, GC, Itabashi-Ku, Tokyo, Japan). Using a pipette, the staff member dispensed stimulated saliva onto a test strip containing three different acid challenges; after 2 min, he or she recorded the colour observed for each test pad. We determined the patient’s buffering capacity by adding the values assigned to each colour, according to the manufacturer’s instructions: green, 4; blue/green, 3; blue, 2; blue/red, 1; and red, 0 (the higher the number, the better the buffering capacity). Hydration from minor salivary glands was measured by assessing the time taken for salivary droplet formation on everted lower lip over 1 min. Subsequently, patient was instructed to chew a piece of paraffin wax for 30 s and expectorate into the spittoon to discard the mixed saliva. The subject continued chewing for 5 min and collected stimulated saliva at regular intervals. The quantity of saliva at 5 min (flow rate) and 1 min was calculated. Stimulated salivary pH was measured by dipping a pH strip in saliva for 20 s and compared to a reference chart (Saliva-Check Buffer, GC, Itabashi-Ku, Tokyo, Japan).

#### Plaque assessment

The Silness and Löe plaque index^41^ was used to assess the presence of plaque. Plaque present in the distofacial, facial, mesiofacial and lingual surfaces near the cervical third of the representative teeth given by the index was evaluated with no intention to assess the middle or incisal thirds. The examination was performed under a light source with a mouth mirror, dental explorer and drying of teeth and gingiva with cotton. Plaque index for each tooth was calculated by adding scores from the four areas of the tooth and then divided by four. By adding the tooth scores together and dividing by the number of teeth examined, the patient’s plaque index (PI) was obtained. Data was categorised based on the scoring criterion: <0.1, no plaque; 0.1–1.0, small quantity of plaque; 1.1–2.0, moderate plaque; and 2.1–3.0, a considerable one.

#### Assessment of dental caries

ICDAS-II 22 was used for visual detection of caries lesions. The teeth were dried and examined by two trained and calibrated dentists using the ICDAS criteria. The sites were recorded as: Code 0 = sound; Code 1 = first visible sign of non-cavitated lesion seen only when the tooth is dry; Code 2 = visible non-cavitated lesion seen when wet and dry; Code 3 = localised enamel breakdown without visual signs of dentine involvement; Code 4 = underlying dark shadow from dentine with or without localised enamel breakdown; Code 5 = distinct cavity with visible dentine; Code 6 = extensive cavitated lesion with visible dentine. A trained dentist then performed oral prophylaxis using prophy paste (Clinpro Prophy Paste, 3M Oral Care, India) and bristle brush with a slow speed hand piece (NSK ER16 16:1 Reduction Contra Angle). Restored teeth without caries were considered normal.

### Reliability of Examination

The oral examination was conducted by trained and calibrated examiner who undertook the online training session on ICDAS assessment. A second calibrated examiner assessed 20% of the screened participants. Interobserver reliability in diagnosing carious lesions was determined using kappa coefficient.

### Statistical Analysis

The data regarding the children affected by dental caries and its risk factors were periodically entered in Microsoft Excel 2010 (V14.0). Data screening and cleaning was performed by the principal investigator. The data was statistically analysed using Statistical Package for the Social Sciences SPSS software version 16.0, SPSS. Threshold was established for various risk factors *a priori*. The ICDAS scores were categorised according to increasing thresholds of dental caries. The definitions for different thresholds were adapted according to International Caries Classification and Management System (ICCMS)^35^ as: scores 0/1, sound tooth structure; 2, initial caries; 3/4, moderate caries; 5/6, extensive caries. Logistic regression with backward exclusion was performed to analyse the risk factors at different thresholds of dental caries (p <0.05). Hosmer and Lemeshow Goodness of Fit test was used to assess the predictability of the regression model. Crude odds ratios with 95% confidence intervals were calculated for statistically significant risk factors.

## Results

### Description of the Population

**Fig 1:**
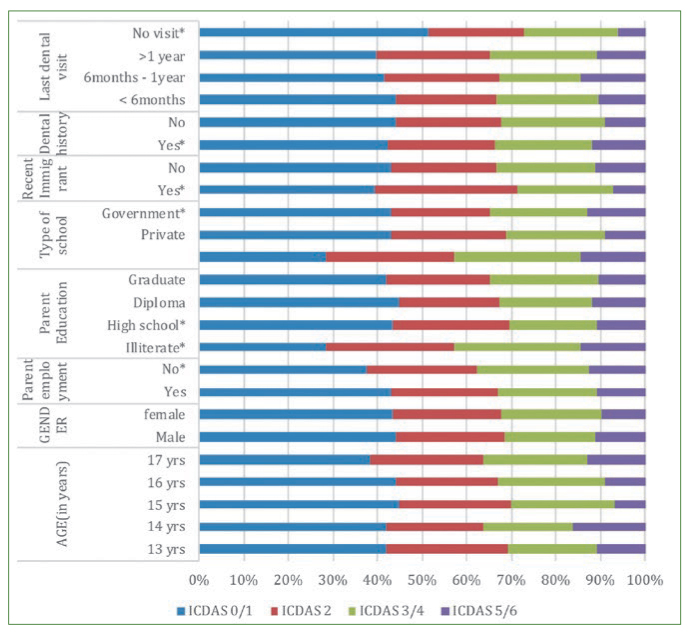
Caries profile of school children based on the demographic details. * Established as negative influence; # Picked up as statistically significant by logistic regression.

**Fig 2:**
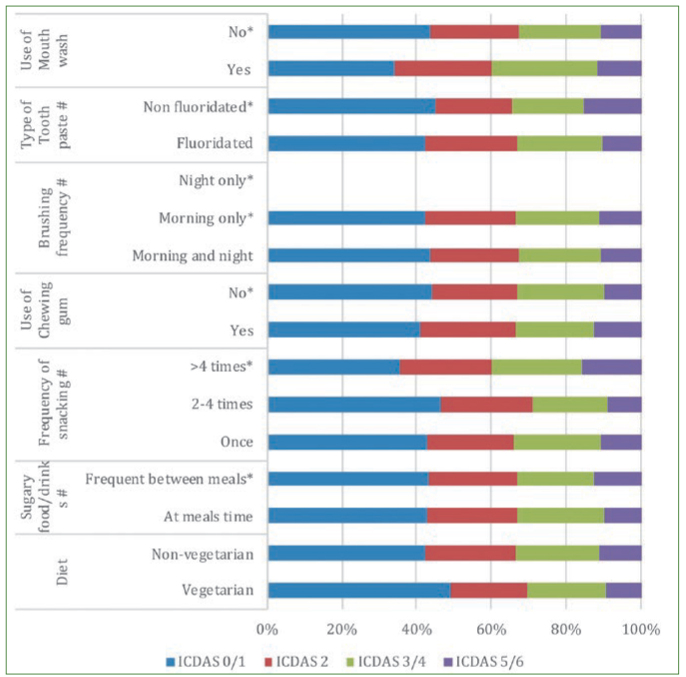
Caries profile of school children based on the habits. * Established as negative influence; # Picked up as statistically significant by logistic regression.

**Fig 3:**
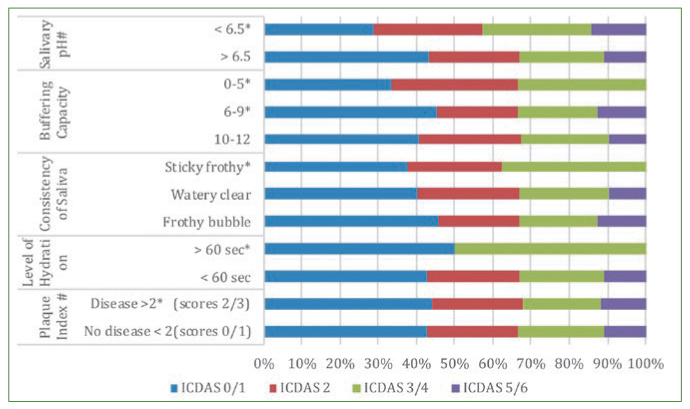
Caries profile of school children based on the clinical parameters * Established as negative influence; # Picked up as statistically significant by logistic regression.

Forty children from five schools each were studied. It was observed that the same child had teeth with different ICDAS scores. Hence it is possible that the same child would be counted multiple times depending upon their caries experience against the established ICDAS thresholds in each row. The caries profile of the school children along with the risk/protective factors and risk indicators are given in Figures 1, 2 and 3. Among the children included in the study, 92 were male and 102 female children with a mean age of 14.9 years.

### Caries Profile

The overall prevalence of dental caries (ICDAS 3–6) was 57.5% (95% CI 48–62%). Prevalence at different thresholds of ICDAS are as follows: initial caries (ICDAS 2): 55% (95% CI 48–62%). Moderate caries (ICDAS 3/4): 51% (95%CI 44–58%). extensive (ICDAS 5/6): 25% (95%CI 19–31%). Interobserver agreement was 0.91.

### Dental Caries and the Associated Factors

Descriptive data for the risk and protective factors are given in Table 1.

**Table 1:** Association of risk factors at different threshold of dental caries

Dental caries threshold	Risk factors	Crude odds ratio	95% Confidence interval crude odds ratio	
			Lower	Upper
ICDAS score 2	Mouthwash	1.40	1.18	1.99
	Quantity of saliva	4.48	2.94	8.23
	Low buffering capacity of stimulated saliva	5.71	2.82	18.2
ICDAS score 3/4	Mouth wash	1.17	1.03	2.09
	Consumption of sugary food	1.81	1.39	2.91
	Quantity of saliva	3.97	2.65	7.03
	pH stimulated saliva	6.24	1.18	32.78
ICDAS score 5/6	pH stimulated saliva	1.73	1.18	1.92

#### Results for ICDAS scores 2 (initial/incipient caries)

**Fig 4:**
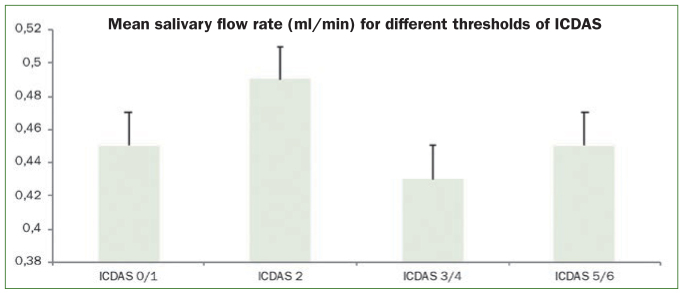
Mean salivary flow rate (ml/min) for different thresholds of ICDAS.

Children of 13 years and 17 years were found to be more prone to development of incipient caries (Fig 1). Low buffering capacity (10–12 points) in stimulated saliva (46.1%) and the mean salivary flow rate (0.49 ± 0.01 ml/min) were associated with the development of incipient lesions (OR 5.71, 95% CI 2.82–18.2 and OR 4.48, 95% CI 2.94–8.23, respectively) (Fig 4). Children with mouthwash use were also prone to developing incipient lesions (OR 1.40, 95% CI =1.18–1.99).

#### Results for ICDAS scores 3/4 (moderate caries)

Children who consumed sugary foods (37.3%) had a higher odds of developing moderate caries (OR 1.81, 95% CI =1.39–2.91) (Fig 2). Among the salivary parameters; salivary flow rate (0.43 ± 0.17 ml/min) and pH (14.7%) were associated with ICDAS score 3/4 (OR 3.97, 95% CI =2.65–7.03 and OR 6.24, 95% CI = 1.18–32.78). The other statistically significant factors associated with the development of ICDAS score 3/4 were use of chewing gums, fluoridated toothpaste (53%). The type of diet which included the sugary foods/drinks (37.3%) (Fig 2). Children using mouthwash were also prone to developing incipient lesions (OR 1.17, 95% CI = 1.03–2.09).

#### Results for ICDAS scores 5/6 (extensive caries)

The odds of developing extensive caries was 1.73 times higher in those children with pH <6.5(24%) (OR 1.73 95% CI = 1.18–1.92). Of those children affected by extensive caries, only 23.5% were using fluoridated toothpaste, and 86.6% of them had the habit of snacking frequently in between meals (Fig 2).

## Discussion

Dental caries is a bacterial-driven, generally chronic, site-specific, multifactorial, dynamic disease process that results from the imbalance in the physiologic equilibrium between the tooth mineral and the plaque fluid. This infectious disease process can be arrested at any point in time.^15^ The high prevalence of dental caries in adolescent children needs focus. Detecting and diagnosing caries lesions at the earliest possible stage are key to arresting disease progression and reversing the demineralisation process.^33^ Nevertheless, despite the marked improvement in oral health, caries occurs in both developed and developing countries where the difference lies in burden of illness. Prevalence in developed countries was found to be 20–29%.^20,36^ In developing countries like India, the high prevalence rates along with the high cost of treatment, with no dental insurance policies, no government (central/state) preventive health policies and much fewer number of water fluoridation facilities unlike other third-world countries renders the population extremely vulnerable.^48^

In our study we chose to estimate the caries profile among the 13–17 years age group as this falls under the vulnerable time period for developing disease in permanent dentition. In India children belonging to this age group are in high school or higher secondary level of education. Age groups 13–14 years and 14.1–15 years were found to be significantly associated with ICDAS score 2 and 5/6, respectively. The reason for this is explained in the WHO manual that all permanent teeth would be erupted and erupted teeth would remain exposed to the risk factors over a considerable time period.^7^

The ICDAS system is based on the ability to visually evaluate the patient’s tooth and restoration conditions with an increased accuracy by providing an easy comparison and interpretation of the studies worldwide.^16^

This system allows the documentation of the severity of lesions.^23^ The International Caries Classification and Management System (ICCMS) is a health outcomes focused system that aims to maintain health and preserve tooth structure. It uses a simple form of the ICDAS Caries Classification model to stage caries severity and assess lesion activity there by defining caries thresholds in order to derive an appropriate, personalised and preventively based, risk-adjusted, tooth-preserving management plan.^34^ The use of ICDAS thresholds for caries profiling showed a distinct difference in our study. The occurrence of dental caries varied with a change in the ICDAS cut off points used to define the disease. ICDAS score 1 was included along with ICDAS score 0 since detecting ICDAS score 1 can be questionable in an epidemiological survey, hence it was considered normal if detected.^12^ The second reason being the lesion is in dynamic phase which is still potentially reversible by chemical means, or arrestable by chemical or mechanical means^11^ and the third, in order to avoid overestimation. ICDAS score 2 was defined as initial/incipient lesion. ICDAS score 3/4 was considered to be moderate caries. ICDAS score 5/6 was considered as extensive caries. Diagnostic threshold for ICDAS score 2 and above correlated with histological sections with good specificity and sensitivity (0.70 and 0.89, respectively), at D3 (ICDAS 2–3) level.^22^

In our study, the questionnaire included several factors like sociodemographic status, immigrant status, personal and dietary behaviour and clinical parameters. Different risk factors were found to be associated with different thresholds of caries. When compared to the normals (ICDAS score 0/1) the consumption of sugary foods/drinks was higher in those children with other thresholds of ICDAS scores (ICDAS 2, ICDAS 3/4). Sugars contained in fermentable carbohydrates, such as milk, juice and starches, are hydrolysed by salivary amylase. This process leads to bacteria-producing acidic end products with subsequent demineralisation of teeth.^26^ Snacking is a major dietary component which is associated with less favourable risk factor profiles. Snacks in India are usually high-fat, high-salt fried foods that may also be high in transfats, and this may explain their relationship with a number of different health outcomes.^29^ It is known that dietary patterns from childhood continue to adulthood.^13,28^ Therefore, knowledge of diet patterns during childhood may provide an opportunity to intervene and prevent chronic diseases. Despite the rapid urbanisation currently occurring in South Asia, diet patterns are likely to differ from those of Western children. Currently, in India, there has been a paradigm shift in diet from a predominantly plant-based, wholegrain diet to a diet high in refined carbohydrates and energy-dense, nutrient-deficient foods and beverages. This shift in the food environment from a ‘traditional’ to a ‘Western’ diet is due to the increased availability and affordability of such foods.^18^ In urban India, over 90% of Indian adolescent participants consume soft drinks. It is estimated that almost all teenagers consume soft drinks and prefer them to healthier drinks, despite the knowledge of problems related to soft drink consumption. Students noted that the home environment provides many opportunities for children to consume soft drinks.^40^

The other protective factors that were significantly associated with ICDAS scores when compared to normals were the use of chewing gums and fluoridated tooth paste which would have enhanced the plaque removal effect and introducing fluoride ions at the clean surface during lesion development phase.^31^ Food additives (polyols) in chewing gums such as xylitol, sorbitol, mannitol, are anticariogenic. The effectiveness comes from salivary flow stimulation through mastication, increase in saliva, biofilm pH and early carious lesion remineralisation.^4^

 The socioeconomic status was found to influence ICDAS thresholds. The factors include the type of school, low family income (may affect the food selection),^43^ the immigrant status (may also affect the degree of education),^35^ the health values, the lifestyles^8^ and the access to healthcare information.^38^

Direct association was found between the quantity/flow of saliva and buffering capacity for all the ICDAS thresholds when compared to normals. It was already studied that the normal level of hydration and higher values for flow rate, pH, buffering capacity of saliva lead to good oral health and a reduced caries occurrence.^23^ Stimulated saliva represents 80–90% of daily salivary production, and the stimulated flow rate varies from 1 to 3 ml/min.^45^ According to a study conducted to assess salivary flow rate, pH, buffering capacity in relation to dental caries severity, age and gender, buffering capacity values were found to be significantly lower in caries active group than in caries-free group.^30^ The most important functions of saliva in preventing caries are its rinsing and buffering effects, in addition to moderating the demineralisation and remineralisation processes by supplying a constant source of calcium and phosphates. Certain salivary parameters such as salivary flow and pH are related to one another. A reduction in salivary flow results in a statistically significant decline in the oral defence systems, which can cause caries and inflammation of the oral mucosa.^47^

A considerable proportion of children who were caries-free were also exposed to the risk factors. This could also be the reason for insignificant association of risk factors to disease state. This phenomenon was classically explained by Geoffrey Rose in his publication titled ‘Sick individuals and sick populations’^37^; where the majority of the population are exposed to a particular risk factor hence could not demonstrate the association of this factor to the disease. If we consider CAMBRA philosophy to the study, half of the population screened in this study face the extreme risk of developing dental caries.^49^ The dental caries status in India is found to be associated with several factors like obesity, oral hygiene and food habits, with an impact on the quality of life. Dental caries in India has not been estimated using ICDAS and hence the severity of the disease is unknown. The present study helps in identifying the proportion of adolescent children with dental caries requiring different treatment needs. The associated factors studied allows targeted preventive measures for this age group.

Low sample size could be a limitation of this study; but we could not include a larger population due to the complexity of risk factors studied. Another limitation was that we could not study stimulated saliva due to lack of cooperation from the children. Due to expected dilution in the oral cavity, the salivary pH cut off was <6.5 instead of the critical pH of 5.5.

## Conclusion

To conclude within the limitations of this study, the proportions of children with incipient caries and moderate caries were high considering their age group. The risk factors associated with incipient caries were different from those associated with moderate and extensive caries. The incipient caries was associated with quantity and low buffering capacity of stimulated saliva, the moderate caries was associated with consumption of sugary food, low salivary flow rate and low pH of stimulated saliva, the severe caries was associated with low pH of stimulated saliva.
